# Novel paradigms for the gut–brain axis during alcohol withdrawal, withdrawal-associated depression, and craving in patients with alcohol use disorder

**DOI:** 10.3389/fpsyt.2023.1203362

**Published:** 2023-09-29

**Authors:** Vatsalya Vatsalya, Joris C. Verster, Manasa Sagaram, Amor J. Royer, Huirong Hu, Ranganathan Parthasarathy, Melanie L. Schwandt, Maiying Kong, Vijay A. Ramchandani, Wenke Feng, Ruchita Agrawal, Xiang Zhang, Craig J. McClain

**Affiliations:** ^1^Division of Gastroenterology, Hepatology and Nutrition, Department of Medicine, University of Louisville, Louisville, KY, United States; ^2^Robley Rex VA Medical Center, Louisville, KY, United States; ^3^National Institute on Alcohol Abuse and Alcoholism, Bethesda, MD, United States; ^4^Clincial Laboratory for the Intervention Development of AUD and Organ Severity, Louisville, KY, United States; ^5^Utrecht Institute for Pharmaceutical Sciences (UIPS), Utrecht University, Utrecht, Netherlands; ^6^Centre for Human Psychopharmacology, Swinburne University, Melbourne, VIC, Australia; ^7^Department of Bioinformatics and Biostatistics, University of Louisville, Louisville, KY, United States; ^8^Alcohol Research Center, University of Louisville, Louisville, KY, United States; ^9^Department of Pharmacology & Toxicology, University of Louisville, Louisville, KY, United States; ^10^Hepatobiology & Toxicology Center, University of Louisville, Louisville, KY, United States; ^11^Seven Counties, Louisville, KY, United States; ^12^Department of Chemistry, University of Louisville, Louisville, KY, United States; ^13^Center for Regulatory and Environmental Analytical Metabolomics, University of Louisville, Louisville, KY, United States

**Keywords:** alcohol dependence (AD), alcohol use disorder (AUD), craving, cytokines, depression, gut–brain axis, heavy drinking, withdrawal

## Abstract

**Introduction:**

Patients with alcohol use disorder (AUD) exhibit symptoms such as alcohol withdrawal, depression, and cravings. The gut-immune response may play a significant role in manifesting these specific symptoms associated with AUD. This study examined the role of gut dysfunction, proinflammatory cytokines, and hormones in characterizing AUD symptoms.

**Methods:**

Forty-eight AUD patients [men (*n* = 34) and women (*n* = 14)] aged 23–63 years were grouped using the Clinical Institute Withdrawal Assessment of Alcohol Scale (CIWA) as clinically significant (CS-CIWA [score > 10] [*n* = 22]) and a clinically not-significant group (NCS-CIWA [score ≤ 10] [*n* = 26]). Clinical data (CIWA, 90-day timeline followback [TLFB90], and lifetime drinking history [LTDH]) and blood samples (for testing proinflammatory cytokines, hormones, and markers of intestinal permeability) were analyzed. A subset of 16 AUD patients was assessed upon admission for their craving tendencies related to drug-seeking behavior using the Penn-Alcohol Craving Score (PACS).

**Results:**

CS-CIWA group patients exhibited unique and significantly higher levels of adiponectin and interleukin (IL)-6 compared to NCS-CIWA. In the CS group, there were significant and high effects of association for the withdrawal score with gut-immune markers (lipopolysaccharide [LPS], adiponectin, IL-6, and IL-8) and for withdrawal-associated depression with gut-immune markers (scored using MADRS with LPS, soluble cells of differentiation type 14 [sCD14], IL-6, and IL-8). Craving (assessed by PACS, the Penn-Alcohol Craving Scale) was significantly characterized by what could be described as gut dysregulation (LBP [lipopolysaccharide binding protein] and leptin) and candidate proinflammatory (IL-1β and TNF-α) markers. Such a pathway model describes the heavy drinking phenotype, HDD90 (heavy drinking days past 90 days), with even higher effects (R^2^ = 0.955, *p* = 0.006) in the AUD patients, who had higher ratings for cravings (PACS > 5).

**Discussion:**

The interaction of gut dysfunction cytokines involved in both inflammation and mediating activity constitutes a novel pathophysiological gut–brain axis for withdrawal symptoms and withdrawal-associated depression and craving symptoms in AUD. AUD patients with reported cravings show a significant characterization of the gut–brain axis response to heavy drinking.

**Trial registration:**

ClinicalTrials.gov, identifier: NCT# 00106106.

## Introduction

Alcohol use disorder (AUD) is a mental health condition that is characterized by heavy and chronic drinking (1). Several pathological mechanisms could be uniquely associated with reward (2), reinforcement (3), cravings (4), withdrawal symptoms (5), and other symptoms of AUD. Most treatment/mechanistic studies have targeted AUD pathophysiology based on the influence of alcohol consumption on brain function measures (6). Recent studies have reported altered gut permeability, gut barrier dysfunction, and increased circulating lipopolysaccharides (LPS, gut-derived bacterial products) due to chronic alcohol consumption and the potentially proximal association of such markers with the behavioral presentation of AUD (7, 8).

Multiple and highly complex gut–brain axis pathways involve brain biochemistry and neuroinflammatory mediators (9). We recently reported altered proinflammatory activity and gut-barrier function in heavy drinkers (10). Excessive alcohol consumption causes altered gut dysbiosis (11), intestinal permeability, and gut-derived inflammation (7, 12, 13). This could be attributed to chronic and heavy drinking patterns (10, 14). These changes result in the translocation of gut/bacterial-derived inflammatory mediators, such as endotoxin (LPS), resulting in gut-derived inflammation (15). Both gut dysfunction and proinflammatory status are commonly encountered with alcohol consumption. Gut-derived inflammation has been postulated to be involved in the neuroinflammation of AUD (16), though the mechanism of gut-barrier dysfunction and proinflammatory response likely constituting a gut–brain axis in AUD is unclear. Thus, understanding the gut–brain axis in the pathology of AUD and its therapy is important.

Chronic and heavy drinking (17–19), as well as frequent episodes of relapse (20–22) observed in AUD, could limit the efficacy of AUD-treatment (23, 24). Alcohol withdrawal is an essential feature of AUD that could predict the rate and frequency of relapse (25). Adiponectin has been studied as a biomarker for withdrawal symptoms and adiponectin's response to the course of withdrawal symptoms, which could have possible links to cravings (26, 27). The role of neuroinflammation in depression has been extensively investigated recently (28, 29). Symptoms of depression are occasionally reported during alcohol withdrawal (11), and the explanation of withdrawal-associated depression could be rooted in the uniqueness of the neuroinflammatory response (30) found in AUD. The role of cravings in alcohol relapse (21) and the involvement of the potential neurocircuitry (31) have been reported recently. Craving for alcohol, along with alcohol withdrawal, is a well-documented risk factor for subsequent relapse in alcohol use disorder (21). Notably, craving is a standalone symptom of AUD as well (32) and could manifest as reward-related behaviors presentation (drinking patterns, scores on AUD-associated questionnaires) (14). The role of the gut–brain axis and proinflammatory activity involved with AUD symptoms such as withdrawal symptoms, depression, and cravings has largely remained understudied so far (33), especially in hypothesis-driven, well-structured clinical investigations. Identifying and characterizing such symptoms will subsequently support precision treatment management for patients with AUD who exhibit such domain/s.

The primary goal of this study was to identify the role of the gut–brain axis and proinflammatory activity in the symptoms of alcohol withdrawal, withdrawal-associated depression, and cravings, as independently observed in AUD patients. We also identified and characterized potential blood biomarkers of gut–barrier dysfunction, endotoxemia, and inflammation involved in the gut–brain axis of AUD. Finally, we evaluated the involvement of sex, age, or other demographics and drinking patterns in the gut–brain axis in patients with AUD.

## Patients and methods

### Patient recruitment

This study was approved by the Institutional Review Board (IRB) of the University of Louisville, Louisville, KY, USA, and the Central Neuroscience (CNS) IRB of the National Institute on Alcohol Abuse and Alcoholism, Bethesda, MD, USA. The study has been indexed on the National Clinical Trials website (clinicaltrials.gov) as NCT00106106. All AUD patients consented to participate in the study before the collection of clinical and research data and bodily samples. A total of 48 patients with AUD, men (*n* = 34) and women (*n* = 14) aged 23–63 years participated in this study. Subjects were diagnosed with AUD as the primary criteria of the study according to DSM-IV, based on the alcohol dependence module of the SCID I-Interview. Participants were considered eligible based on the following criteria: (1) clinically manifested significant alcohol withdrawal, with or without detectable blood alcohol concentrations (BACs); or (2) in the absence of the above, a current intoxication level >0.1 g/dl BAC, and a self-reported history of continuous alcohol intake for more than the past 1 month (as well as self-reported previous episodes of significantly distressful alcohol withdrawal). More information on patient participation and enrollment can be obtained in previous publications (17, 34, 35).

### Clinical and research data

Forty-eight enrolled study participants were grouped categorically using the abbreviated version of Clinical Institute Withdrawal Assessment of Alcohol Scale (CIWA-Ar) into clinically significant CIWA groups (CS-CIWA [score >10] [*n* = 22]), and a clinically not-significant group (NCS-CIWA [score ≤ 10] [*n* = 26]) (36). We used ≥10 as eligibility criteria for a diagnosis based on mild and above-average severity of withdrawal symptoms for identifying withdrawal status (http://www.regionstrauma.org/blogs/ciwa.pdf). Clinical data and blood samples were collected upon enrollment. Demographics (age, sex, and body mass index [BMI]) and recent drinking history information were also recorded at the time of the study visit. CIWA (36) scores, Montgomery-Asberg Depression Rating Scale (MADRS) (37) assessment, craving assessment (Penn Alcohol Craving Scale [PACS]) (38), 90-day timeline followback (39) (TLFB90: TD90, NDD90, AvgD90, HDD90) for recent drinking profile, lifetime drinking history (LTDH) (40), and alcohol composite score [CS, from the Addiction Severity Index assessment (ASI) (41)] were also collected for analyses. MADRS criteria for reference ranges were used [Montgomery-Asberg Depression Rating Scale (MADRS)—MDCalc] from previously reported publications (37, 42). PACS was performed on a subset of 16 AUD study patients (at intake, regardless of the presence/absence of withdrawal symptoms); PACS was not collected on all the participants of this study. Its association with the markers of gut alterations, heavy drinking, and inflammation was assessed. PACS ≥ 6 was used for identifying drinking levels and the cytokine/gut-permeability measures that might predict moderate to high cravings (43).

Information on recent drinking and patterns of drinking was collected from the Timeline Followback Questionnaire (39), a self-reported inventory of alcohol intake that was collected in the clinical setting under clinical supervision. Measures were assessed for the past 90 days and included total drinks (TD90), number of drinking days (NDD90), number of non-drinking days (NNDD90), average drinking per drinking day (AvgDPD90), and heavy drinking days (HDD90). Chronic drinking (as the number of years) was assessed using the LTDH (in years) questionnaire (40). We also used the “Controlling Nutritional Status Test” (CONUT) to establish the nutritional status (44) of our patients.

### Blood samples and laboratory testing

Blood samples were collected during the study visit for each enrolled patient. Blood samples were processed for plasma and frozen at −80°C until assayed. Plasma samples were analyzed for the proinflammatory cytokines (interleukin-6 [IL-6], interleukin-8 [IL-8], tumor necrosis factor-α [TNF-α], interleukin-1β [IL-1β], and monocyte chemoattractant protein-1 [MCP-1]), adiponectin, and leptin by multianalyte chemiluminescent detection using Multiplex kits (Millipore, Billerica, MA, USA) on the Luminex platform (Luminex, Austin, TX, USA) based on the manufacturers' instructions. Plasma LPS and LPS binding protein (LBP) and soluble cells of differentiation type 14 (+sCD14) were assessed using the Kinetic Chromogenic Limulus Amoebocyte Lysate Assay (Lonza, Walkersville, MD, USA) following the manufacturer's instructions.

### Statistical analyses

A univariate factorial ANOVA was used to evaluate the group differences in the demographic and drinking history markers, scores of CIWA, and other alcohol-associated symptoms such as depression, gut-permeability measures, and levels of various cytokines. The primary factor used to group the patients was the presence or absence of clinically significant withdrawal symptoms. A univariate ANOVA was used for coding to evaluate these differences, with an additional discrete secondary factor of sex as a between-group response and statistical interaction for sub-set assessments. Sex-based association effects within each sex were also evaluated as per the scope. Drinking history and other demographic factors were tested as confounders of inflammation in AUD. A linear regression analysis was used to characterize the association between drinking symptoms, drinking measures, and laboratory biomarkers. The laboratory markers of AUD symptoms, such as hormones and proinflammatory cytokines (adiponectin, IL-1β, IL-6, IL-8, TNF-α, MCP-1, leptin, and other proinflammatory cytokines and hormones), were tested for the within-group associations with the clinical and drinking markers and with gut-permeability measures (LPS, LBP, and sCD14) using the linear regression analysis (as univariate or multivariate independent variable models).

**Figure 1:**
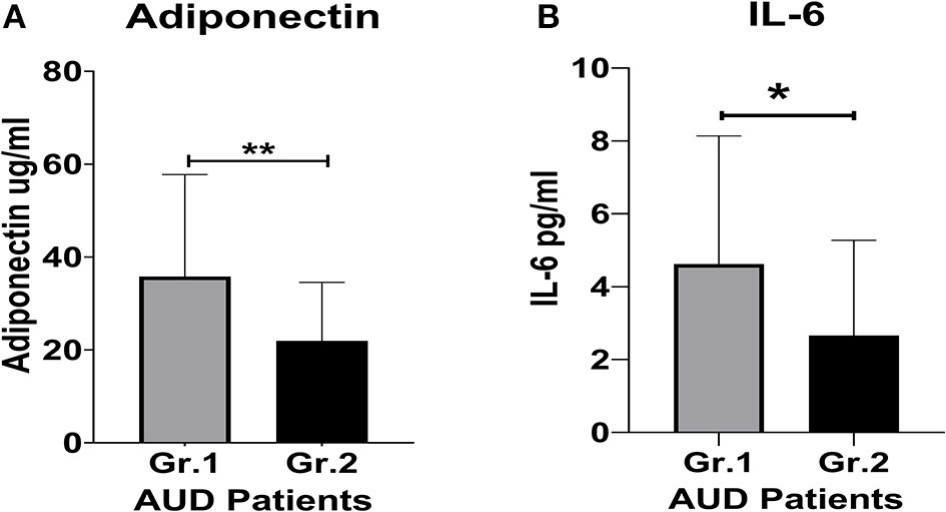
Proinflammatory cytokine and protein hormone activity in AUD patients grouped by clinically significant (CS) level of alcohol withdrawal identified using CIWA scores. **(A)** Adiponectin levels. **(B)** IL-6 levels. Data presented as M±SD. Statistical significance was set at *p* < 0.05. **p < 0.01. *p < 0.05.

**Figure 2:**
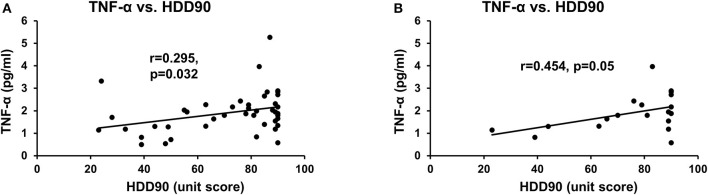
Association of heavy drinking and proinflammatory activity. **(A)** TNF-α and HDD90 in all AUD patients. **(B)** TNF-α and HDD90 in CS-AUD patients with clinically relevant withdrawal scores. r denotes the correlation coefficient of the relation. Statistical significance was set at *p* < 0.05.

**Figure 3:**
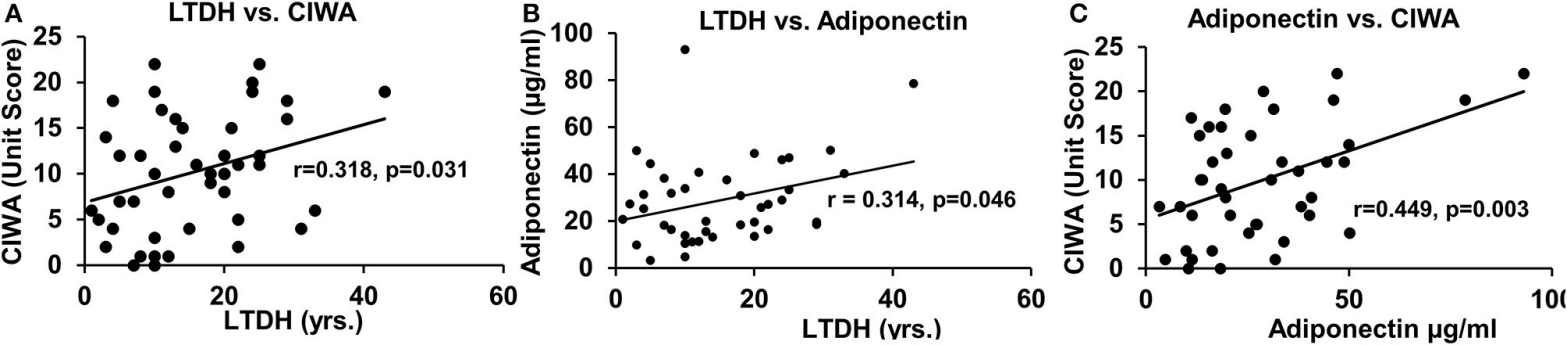
Association of withdrawal score with chronic drinking measures and levels of adiponectin in all the AUD patients. **(A)** Association of chronic drinking (LTDH) and CIWA. **(B)** Association of chronic drinking (LTDH) and adiponectin. **(C)** Association of CIWA and plasma adiponectin level (A laboratory marker of withdrawal symptoms). Statistical significance was set at *p* < 0.05.

**Figure 4:**
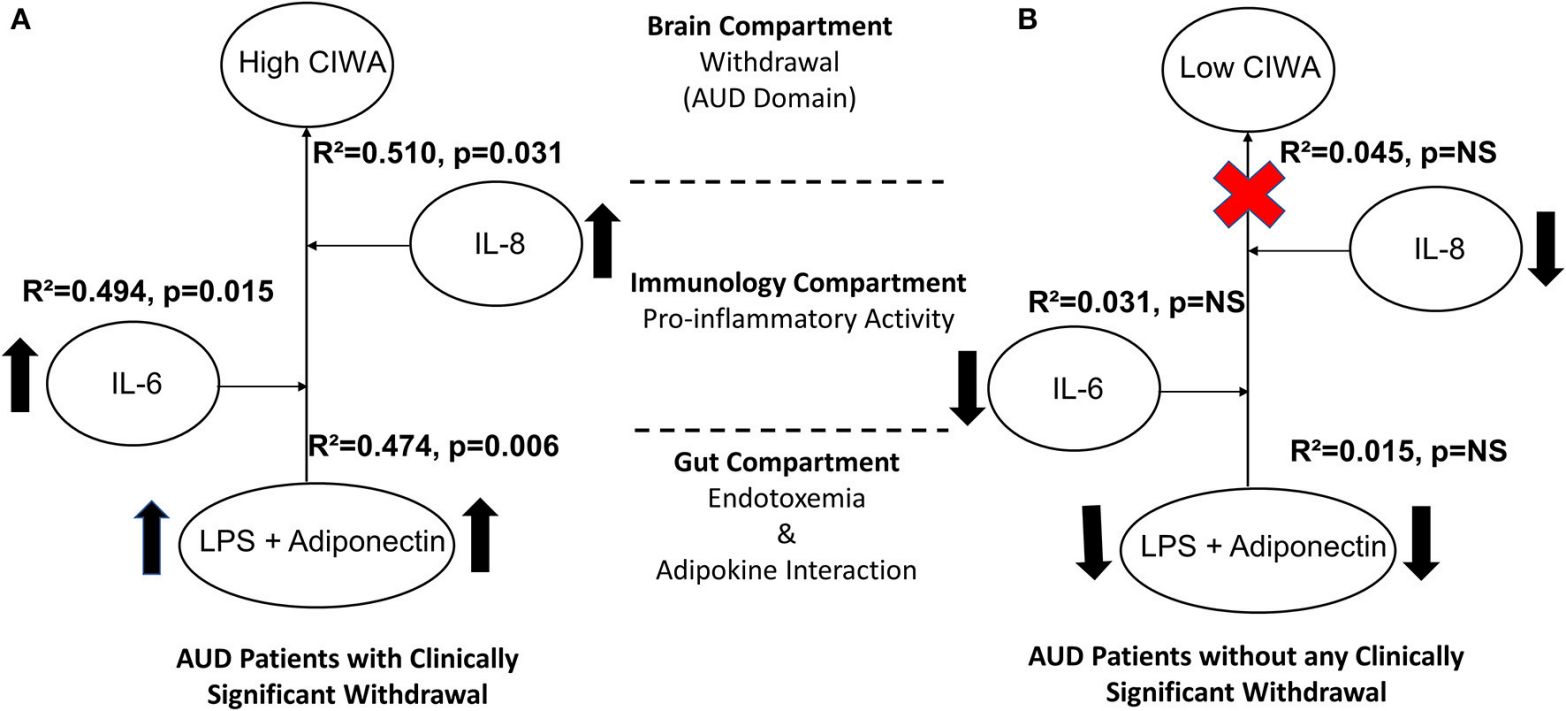
Model for the gut–brain axis illustrating effects and schema of gut dysfunction, proinflammatory, and adiponectin activity on alcohol withdrawal in **(A)** AUD patients with clinically significant CIWA scores and **(B)** patients without clinically significant withdrawal symptoms. Statistical significance was set at *p* < 0.05. Arrows indicate higher levels of specific measures observed in CS than in the NCS group. Upward arrows show a comparative increase between the two sub-figures. The red cross sign denotes no effect. Effect size ranges: mild <0.2; 0.2<moderate>0.7; robust>0.7.

**Figure 5:**
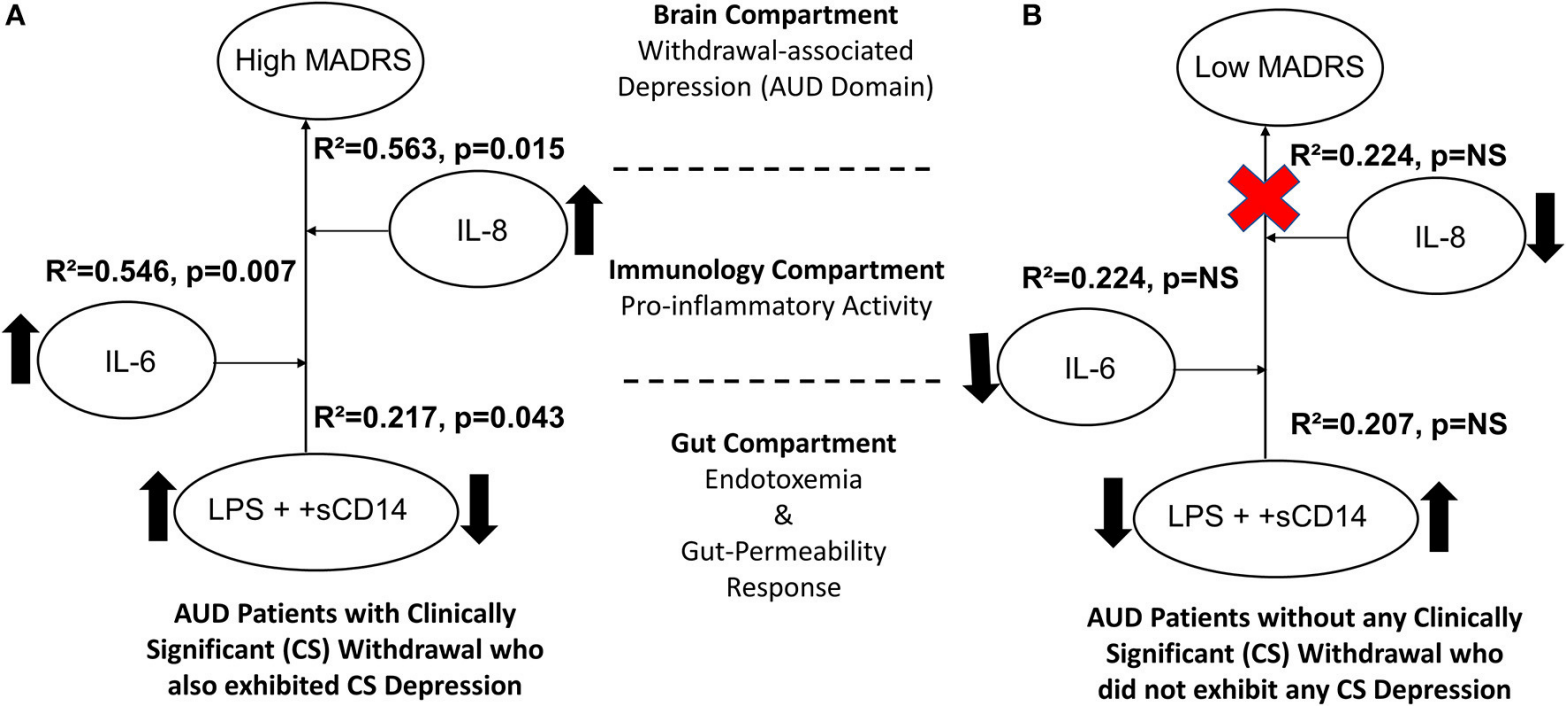
Model for the gut–brain axis involving gut-dysfunction proinflammatory activity on MADRS scores in **(A)** AUD patients with clinically significant withdrawal symptoms (CS) and **(B)** AUD patients without clinically significant withdrawal symptoms (NCS). Statistical significance was set at *p* < 0.05. Upward arrows indicate higher levels of specific measures observed in CS than the NCS values. The red cross sign denotes no effect. Effect size ranges: mild <0.2; 0.2<moderate>0.7; robust>0.7.

**Figure 6:**
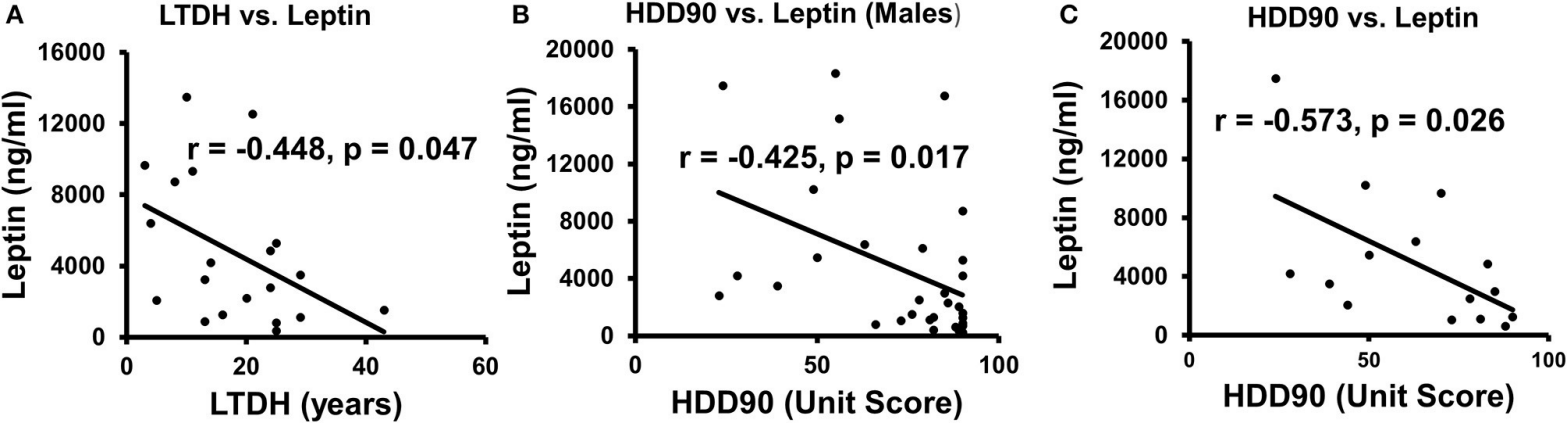
Association of patterns of alcohol intake and leptin in AUD patients. **(A)** Lifetime drinking history (LTDH) chronic drinking markers showed a negative association with leptin in CS-AUD patients exhibiting withdrawal symptoms. **(B)** Recent heavy drinking patterns in the past 90 days (HDD90) showed a negative relationship with leptin in this study's male AUD patients. **(C)** HDD90 and leptin showed a higher negative association among the AUD patients who reported on the PACS score. Statistical significance was set at *p* < 0.05.

Individual gut–brain axis models specific for each AUD domain of this study were constructed for withdrawal symptoms and depression, including immunological status. A multivariate stepwise predictive model was used using SPSS software using a multivariate regression model with stepwise addition of variable(s) sequenced by the level of compartmentalization of the gut-immune-brain pathway (primary independent variables belonging to the altered markers of the gut compartment; intermediate as immune and resulting in a dependent variable as the output). A predictive model for a sub-set of the study patients was also developed for characterizing the gut–brain axis of cravings based on the PACS score using a multivariate regression model with stepwise addition of variable/s contributing at various steps of the gut–brain pathway. The PACS score was also used as a factor to assess the prediction model of heavy drinking patterns and candidate gut-immune measures. SPSS 27.0 (IBM, Chicago, IL, USA) and the Microsoft 365 Excel application (MS Corp., Redmond, WA, USA) were used for statistical and analysis data computation. GraphPad Prism was used to build Figures 1–3, 6, and Supplementary Figure 1 was made in MS 365. Figures 4–6 were developed in MS PowerPoint. Statistical significance was established at *p* ≤ 0.05. Data are expressed as M ± SD (mean ± standard deviation) unless otherwise specified.

## Results

### Demographics, drinking, and nutritional assessment

Demographic measures (age and BMI) were similar in both study groups and showed no remarkable numerical or statistical difference. The CIWA score in the CS (with clinically significant [CS] CIWA scores) patients (45.34% of the overall study participants) was 3-fold higher than in NCS (clinically not significant) patients (Table 1), as expected. Moreover, as expected, all heavy drinking markers were numerically higher in the CS group. Notably, the HDD90 and NDD90 (based on the TLFB90 assessment), along with the LTDH (longitudinal/chronic drinking) drinking markers, were significantly higher in the CS group (Table 1). Additionally, the men in the CS group had significantly higher LTDH levels (*p* = 0.050) compared to women. However, recent drinking assessments revealed that women in the CS group exhibited more severe patterns of alcohol consumption (Table 1). We found no other sex differences in drinking markers in the CS group.

**Table 1:** Demographics, markers of recent and long-term drinking history, withdrawal score, and gut permeability measures in AUD patients grouped by the presence of clinically relevant withdrawal symptoms.

**Measures**	**CS-CIWA**			**NCS-CIWA**			**Between-group *p*-value**
	**Men (13)**	**Women (9)**	**Total (22; 45.8%)**	**Men (21)**	**Women (5)**	**Total (26; 54.2%)**	
Age (years)	43.97 ± 11.9	41.25 ± 10.6	43.1 ± 11.2	43.28 ± 9.2	43.14 ± 12.9	43.25 ± 9.8	NS
BMI (kg/m^2^)	25.81 ± 3.9	30.87 ± 7.7	27.49 ± 5.8	26.69 ± 4.5	26.76 ± 8.7	26.71± 6.0	NS
CONUT	1.15 ± 1.4	0.78 ± 1.0	1.00 ± 1.2	1.00 ± 1.2	0.80 ± 0.8	0.96 ± 1.1	NS
**Drinking profile and alcohol-associated measures**							
TD90	1,206.9 ± 585.1	1,368.0 ± 659.6	1,260.6 ± 595.4	1,202.0 ± 600.6	632.5 ± 296.0	1,072.5 ± 592.6	*0.081*
HDD90	75.08 ± 22.4	83.34 ± 7.8	77.83 ± 18.9	71.65 ± 22.0	54.0 ± 21.6	67.64 ± 22.7	0.033
AvgDPD90	16.32 ± 6.6	16.18 ± 7.8	16.27 ± 6.8	15.17 ± 5.6	12.5 ± 8.1	14.55 ± 6.2	NS
NDD90	76.83 ± 22.4	83.50 ± 7.7	79.06 ± 18.8	76.17 ± 17.5	56.2 ± 21.9	71.66 ± 19.9	0.044
ACS	0.82 ± 0.1	0.81 ± 0.1	0.82 ± 0.1	0.83 ± 0.1	0.66 ± 0.2	0.80 ± 0.2	NS
LTDH^a, b, d^	21.0 ± 10.7	13.67 ± 7.7	18.56 ± 10.2	15.65 ± 8.7	6.0 ± 2.0	13.45 ± 8.7	0.045
MADRS	16.58 ± 7.9	21.11 ± 6.2	18.52 ± 7.5	14.22 ± 7.9	13.2 ± 9.0	14.00 ± 7.9	*0.058*
DCS	0.037 ± 0.08	0.038 ± 0.09	0.038 ± 0.08	0.059 ± 0.1	0.061 ± 0.1	0.060 ± 0.1	NS
CIWA^d^	15.46 ± 3.8	15.89 ± 3.6	15.64 ± 3.6	5.67 ± 3.2	3.40 ± 3.4	5.23 ± 3.3	NA
PACS	7.25 ± 7.9	11.0 ± 14.2	8.86 ± 10.1	11.0 ± 4.3	–	11.0 ± 4.3	NS
**Gut-permeability and proinflammatory measures**							
LPS (EU/ml)	0.109 ± 0.06	0.114 ± 0.07	0.111 ± 0.06	0.095 ± 0.06	0.05 ± 0.03	0.089 ± 0.06	NS
LBP (pg/ml)	2,601.19 ± 3,369.2	2,305.07 ± 3,942.1	2,492.09 ± 3,484.03	1,115.33 ± 1,241.9	2,508.30 ± 3,980.3	1,383.21 ± 2,020.42	NS
+sCD14 (pg/ml)^a^	8,420.4 ± 1,514.2	10,075.19 ± 1,140.1	9,097.4 ± 1,580.8	9,255.16 ± 1,974.6	10,403.67 ± 1,991.5	9,476.03 ± 1,991.7	NS

AUD patients are categorized by sex within each of the primary groups. CS, Clinically significant group; NCS, clinically not significant group; BMI, body mass index; CONUT, controlling nutritional status; TLFB90, timeline follow back past 90 days: markers (recent drinking history [unit score]) (1) TD90: total drinks in 90 days; (2) AvgDPD90: average drinks per drinking day in the last 90 days; (3) HDD90: heavy drinking days in the last 90 days; (4) NDD90: number of drinking days in the last 90 days; ACS, alcohol composite score; LTDH, lifetime drinking history (retrospective drinking history of life); MADRS, Montgomery-Asberg Depression Rating Scale; DCS, Drug Composite Score; CIWA, CIWA score (Unit score) for withdrawal scale; PACS, Penn-Alcohol Craving Scale; LPS, Lipopolysaccharide; LBP, Lipopolysaccharide-binding protein; +sCD14, Soluble Cluster of Differentiation 14; NS, Not Significant; NA, Not Applicable. P-value in italics: trend-level response. ^a^sex-difference within Group 1. ^b^sex-difference within group 2. ^c^within male-sex differences between the two groups (none determined). ^d^within female-sex differences between the two groups.

The women in the CS group consumed more than twice as much alcohol in the past 90 days and over the past number of years compared to the women in the NCS group (Table 1). The women in the CS group also had 37.5% higher Montgomery-Asberg Depression Rating Scale (MADRS) scores and a significant ~5-fold higher CIWA scores compared to the women in the NCS group. The men in the CS group exhibited ~3-fold elevated CIWA scores compared to the men in the NCS group. On the other hand, the men in the NCS group drank almost twice the amount that the women in the NCS group drank in the past 90 days (there was a trend of higher volume of drinking, TD90, *p* = 0.052), as well as of NDD90 in the NCS group men vs. women (*p* = 0.056). LTDH and Alcohol Composite Score (ACS) were significantly higher in the men of the NCS group compared to their female counterparts, as well (Table 1). There were no differences in the CONUT scores between the two groups or sex differences within each group.

### Withdrawal markers, proinflammatory activity, and gut-dysfunction response

Adiponectin, a biomarker for alcohol withdrawal (26), was significantly (*p* = 0.013) elevated in the CS group. The increase was also approximately 2-fold higher compared to the NCS group (Figure 1A). The women in Group 1 exhibited ~37% higher adiponectin levels than the men in the CS group (Table 1). Importantly, the women in the CS group also had more than 2-fold higher adiponectin levels than the women in the NCS group (Table 1). This difference was not as well-defined in the men.

The CS group had a 2-fold higher IL-6 level compared to the NCS group (Figure 1B). Notably, in the CS group, the women exhibited a ~57% higher level of IL-6 than their male counterparts (Table 1). The women in the CS group showed numerically higher IL-8 levels (trend level of significance, *p* = 0.070) than the men in the CS group (Table 2); however, due to huge variability, it did not reach statistical significance.

**Table 2:** Candidate proinflammatory cytokines and hormones in AUD patients grouped by the presence of clinically relevant withdrawal symptoms.

**Measures**	**CS-CIWA**			**NCS-CIWA**			***Between-group p*-value**
	**Men (13)**	**Women (9)**	**Total (22; 45.8%)**	**Men (21)**	**Women (5)**	**Total (26; 54.2%)**	
IL-6 (pg/ml)	3.77 ± 2.2	5.92 ± 4.8	4.62 ± 3.5	2.70 ± 2.7	2.52 ± 2.7	2.66 ± 2.6	*p =* 0.039
TNF-α (pg/ml)	1.80 ± 0.8	2.02 ± 0.9	1.89 ± 0.8	1.90 ± 0.6	1.95 ± 2.0	1.91 ± 1.0	NS
IL-1β (pg/ml)	0.52 ± 0.3	0.48 ± 0.6	0.51 ± 0.4	0.52 ± 0.3	0.56 ± 0.4	0.52 ± 0.3	NS
MCP-1 (pg/ml)^a^	110.03 ± 59.9	98.37 ± 39.1	105.37 ± 51.7	100.14 ± 31.4	182.3 ± 121.4	117.3 ± 67.0	NS
IL-8 (pg/ml)	4.49 ± 2.6	20.16 ± 28.4	10.76 ± 19.1	4.28 ± 3.3	5.96 ± 7.2	4.61 ± 4.2	NS
Leptin (pg/ml)	3,074.5 ± 2,631.7	7,179.5 ± 4,651.3	4,716.5 ± 4,029.5	5,766.5 ± 6,419.2	10,125.2 ± 10,752.4	6,674.5 ± 7,458.2	NS
Adiponectin (μg/ml)	30.95 ± 19.1	42.5 ± 25.2	35.79 ± 22.0	22.36 ± 12.5	20.32 ± 14.6	21.93 ± 12.6	*p =* 0.013

AUD patients are divided by sex within each of the primary groups. CS, Clinically Significant group; NCS, Clinically Not Significant group; CIWA, Clinical Institute Withdrawal Assessment Alcohol Scale; NS, Not Significant; NA, Not Applicable. *P*-value in italics: trend-level response. ^a^sex-difference within group 2.

Gut permeability markers (especially LBP) were numerically higher in the CS group (albeit did not reach statistical significance, likely due to a larger standard deviation in both groups, Table 1). LBP was ~2.3-fold higher in the men in the CS group compared to the men in the NCS group (There was a trend level of statistical significance, *p* = 0.077). The women in the CS group showed significantly higher +sCD14 levels than their CS group male counterparts (Table 1).

In the CS group, LBP was significantly associated with heavy drinking patterns, specifically HDD90 (R^2^ = 0.351 *p* = 0.010); *p* = 0.022 for the association).

HDD90 and TNF-α values showed a statistically significant, albeit mild, correlation across all the study patients (Figure 2A). However, the underlying effect of this association was due to the modest effect size between HDD90 and TNF-α (R^2^ = 0.207), which was significant (r = 0.454 at *p* = 0.050) but limited in the CS group (Figure 2B). Assessment of such an association was not significant in the NCS group.

### Alcohol withdrawal, drinking patterns, and gut–brain axis markers

The withdrawal score CIWA and adiponectin were significantly associated with the chronic drinking marker LTDH in all patients with AUD (Figures 3A, B, respectively). CIWA was also significantly associated with adiponectin in all AUD patients (Figure 3C).

To establish the gut–brain axis in our cohort for withdrawal symptoms, we used a multivariable regression model. We tested adiponectin and LPS as predictors for gut dysfunction, IL-6, and IL-8 for proinflammatory activity. CIWA (a continuous dependent variable) was mildly associated with LPS, adiponectin, IL-6, and IL-8 at R^2^ = 0.279 at *p* = 0.013. Further, we independently used the same statistical model for the CS and NCS groups. Only the CS patients showed a stepwise increase in the strength of the association between CIWA scores, adiponectin, and LPS (R^2^ = 0.474, *p* = 0.006). The effect size of this association augmented further with respect to proinflammatory activity in a stepwise manner for IL-6 (R^2^ = 0.494, *p* = 0.015) and IL-8 (R^2^ = 0.510, *p* = 0.031) (Figure 4A, schematic). The patients in the NCS group did not exhibit such an arrangement of gut-immune-brain responses (Figure 4B).

### Depression associated with alcohol withdrawal, drinking patterns, and gut–brain axis markers

The MADRS score was significantly associated with +sCD14 response (a gut-dysfunction measure that facilitates cellular responses to LPS) with mild effects (R^2^ = 0.208 at *p* = 0.038) in the CS group that were augmented (R^2^ = 0.217 at *p* = 0.043) in conjunction with the confounder LPS (circulating LPS indicates gut barrier dysfunction/leakiness). Ultimately, this arrangement of gut dysfunction on MADRS showed much higher effects in the context of candidate proinflammatory activity in a stepwise increasing fashion, with IL-6 (R^2^ = 0.546, *p* = 0.007) and IL-8 (R^2^ = 0.563, *p* = 0.015), respectively (Figure 5A). Increased levels of endotoxemia-associated proinflammatory cytokine responses were evident. Notably, there were significantly higher levels of +sCD14 in women than men in the CS group. Such a strong gut–brain axis signature on the depression domain was not present in the NCS group (Figure 5B).

### Craving and leptin response, drinking patterns, and gut–brain axis markers

Leptin levels in the CS group, a marker of cravings (45), were lower, approximately 2/3 times those of the NCS group levels (Table 2). An inverse relationship between LTDH and leptin levels was significant only in the patients in the CS group (R^2^ = 0.201, *p* = 0.047) (Figure 6A). In the NCS AUD patients without any withdrawal symptoms, higher levels of leptin and lower HDD90 values showed a significant association *p* = 0.038 at mild effects, R^2^ = 0.14; this association was not significant in the patients in the CS group. Notably, leptin and HDD90 showed a significant inverse, albeit mild, association exclusively among the men of this study (regardless of withdrawal symptoms), R^2^ = 0.181 at *p* = 0.017 (Figure 6B). Leptin levels also showed sexual dimorphism, showing a particular vulnerability in men with AUD (46) (especially those in the CS group who had clinically significant withdrawal symptoms). These values were less than half of those found in women of the same CS group at *p* = 0.021 (Table 2).

A subset analysis involving 16 AUD patients was conducted to assess cravings. This analysis revealed unique and significant gut–brain responses, as well as the involvement of proinflammatory cytokines when assessed using PACS scores. There were 13 men and 3 women who did not show much numerical or statistical sex difference in PACS scores. AUD patients, whose cravings were recorded using PACS scores, showed a significant inverse association between leptin and HDD90, R^2^ = 0.328 at *p* = 0.026 (Figure 6C). PACS was also significantly and positively associated *p* = 0.011 (R^2^ = 0.40) with the gut-permeability marker LBP.

**Figure 7:**
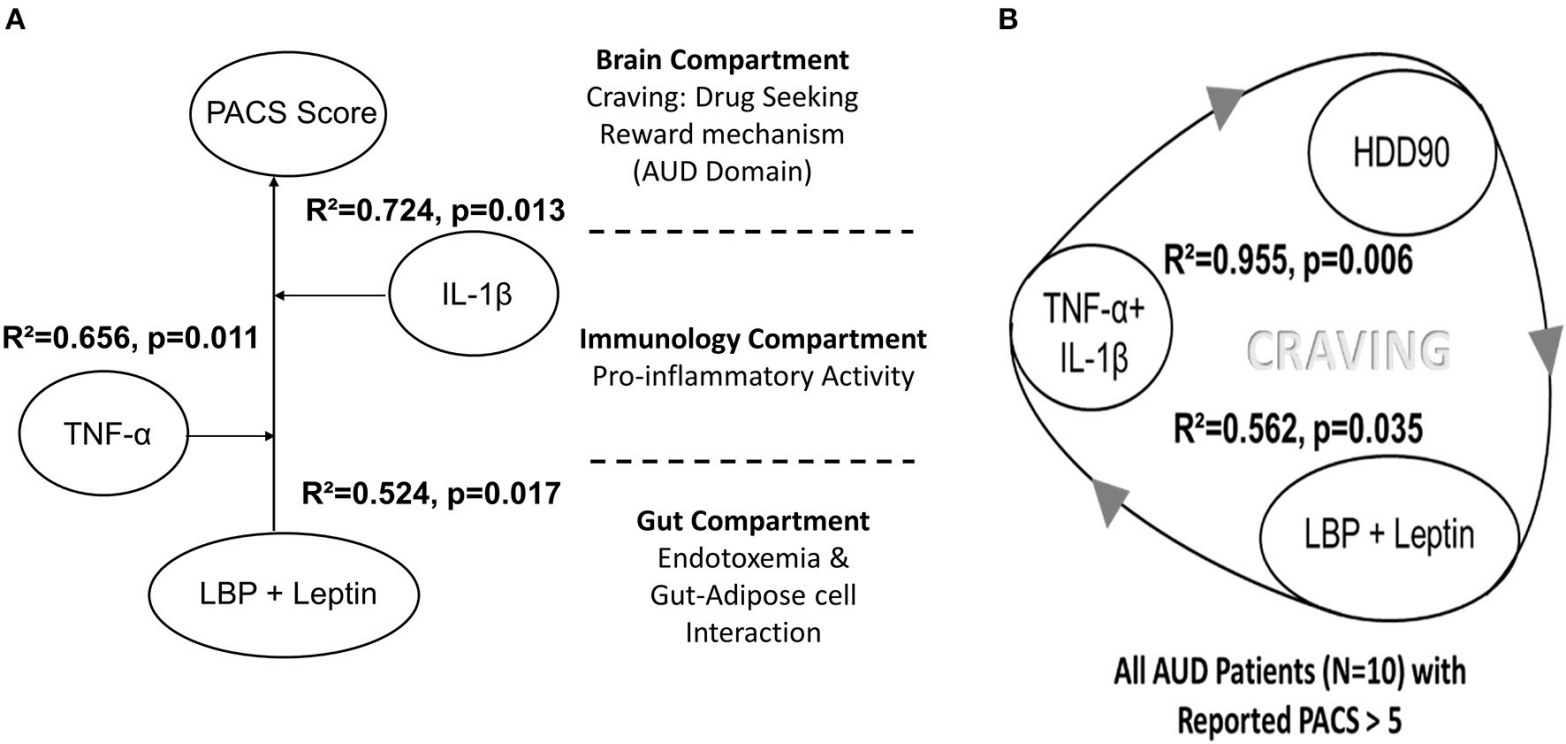
**(A)** Model for the gut–brain axis involving gut-dysfunction proinflammatory activity on the craving scale (PACS) in a sub-set of AUD patients in this study. **(B)** The corresponding model for heavy drinking days (HDD90), a marker of heavy drinking, exhibited very high effects in AUD patients who exhibited PACS > 5 (*N* = 11, though the model was run on 9 patients who had data for all the variables). Statistical significance was set at *p* < 0.05. Effect size ranges: mild <0.2; 0.2<moderate>0.7; robust>0.7.

We evaluated the gut-immune-brain connection, specifically in the context of craving. Our focus was to identify the role of LBP and leptin in gut dysfunction, as well as TNF-α and IL-1β in proinflammatory immunological status. This combination of gut dysfunction and proinflammatory markers had a significant impact, strongly predicting the PACS score (Figure 7A). When we applied the same gut–brain model to evaluate the drinking response, as measured by the heavy drinking marker, HDD90, we found an even higher effect size (Figure 7B) in AUD patients with higher cravings (PACS > 5, *n* = 11, model tested on *n* = 9 with complete data on all variables). This model was tested on 9 patients who had complete data on all variables. Interestingly, this association was not observed in patients with PACS scores <5. A highly robust effect size at significant levels showed high reproducibility and a closely fitted arrangement of unique gut dysfunction and immune responses that are both triggered by heavy drinking. In return, such patients keep drinking heavily and report higher cravings, likely spiraling into a vicious cycle.

## Discussion

Our study revealed that AUD patients with clinically significant CIWA scores and symptoms of withdrawal-associated depression and craving symptoms presented with exacerbated patterns of heavy and chronic drinking. The unique interplay between heavy and chronic drinking, gut dysfunction, and proinflammatory response levels reveals a complex cascade. This cascade characterizes various aspects of AUD, including specific withdrawal symptoms, depression, and cravings. Several gut-dysfunction biomarkers were detected that corresponded well with the changes in cytokine concentrations, indicating proinflammatory activity. The gut-immune changes ultimately characterize the phenotypic (either exogenous as clinical symptoms [behavioral responses and observations/assessments] constituting the clinical symptoms and/or endogenous as research/clinical laboratory markers) presentation of AUD.

### Role of alcohol consumption in the gut–brain axis of AUD

The study has clearly shown that excessive alcohol consumption leads to both increased circulating levels of LPS derived from gut bacterial products and increased gut permeability (47). Subsequent dysregulation in gut permeability and function that we addressed in the context of LPS and other proinflammatory changes (increase in adiponectin, cytokines, etc.) could follow up with the establishment of the proinflammatory status. Alteration in immune response with alcohol consumption leads to proinflammatory status (48). The drinking profiles of the study patients exhibited uniquely heavy drinking patterns, especially HDD90, NDD90, and TD90. These acutely elevated drinking patterns suggest remarkable changes in drinking behavior reported in the past 90 days.

Under normal circumstances, the human gut maintains its microbiome (49). Functional changes in this commensal microbiota led to several pathogenetic conditions. We identified a corresponding increase in the level of withdrawal symptoms with lifetime drinking. Several findings suggest that changes in the gut microbiome (dysbiosis) largely contribute to certain deleterious effects of chronic excessive alcohol intake (50, 51), and attenuating these changes is a therapeutic option that has great interest (52). These reports revealed an important biological association between gut dysregulation and alcohol intake, emphasizing the need for therapeutic intervention on gut microbial targets to treat AUD.

### Key findings on sex differences

We found that the female AUD patients with clinically significant withdrawal symptoms drank more heavily than their male counterparts (with similar withdrawal scores), suggesting that they drink at levels that might go unnoticed if overall drinking is only considered. Women exhibit more severe heavy drinking patterns (53), which can be corroborated by the fact that women can develop the clinical presentation of AUD even with a shorter duration of reported drinking. Thus, the severe form of heavy drinking observed among women facilitates a rapid pathological course (including other predispositions (54) among the women) (55), reaching a comparable clinical presentation (56). Importantly, the ongoing adverse influence on other symptoms of AUD and alcohol-associated organ injury may well be progressing simultaneously, either independently or in a comorbid form. Evidently, we found that this group of female patients also showed correspondingly higher MADRS scores (depression symptoms). These findings may be related to the sex-specific vulnerability of female patients, more so when compared with their male counterparts (19, 57, 58). We also want to mention that in the CS group, the women with withdrawal symptoms showed a more weakened immune activity response.

### Assessment of the gut–brain axis in the context of withdrawal symptoms

Patients with AUD exhibiting withdrawal symptoms exhibited a close association between proinflammatory cytokines and adiponectin. The interaction of adiponectin and LPS and higher proinflammatory cytokine levels supports the role of proinflammatory activity in the context of withdrawal symptoms and a possible pathogenic role of the gut–brain axis in exacerbating withdrawal symptoms. In our study, women with high withdrawal symptoms showed a greater predisposition (57), indicated by changes in gut permeability and proinflammatory cytokine responses. Previous studies showed adiponectin levels (7.94 μg/ml) in patients with moderate alcohol drinking histories (59). Our study on heavy drinkers with withdrawal symptoms showed adiponectin levels that were approximately five times higher than those without withdrawal symptoms, which nonetheless were 2.5 times higher. Increased adiponectin levels have also been reported as a possible link to cravings in alcohol-dependent patients, specifically male patients (27). There could be a relationship between the higher levels of adiponectin that we report as an outcome of higher chronic alcohol drinking and the positive, strong association that we discussed between adiponectin and lifetime drinking years. Adiponectin may play an important role in metabolic signaling (especially after binding to AdipoR1), activating various downstream events associated with adiponectin function (such as AMP-activated protein kinase, etc.) (60). Since the assessments of adiponectin were performed at admission among the patients, the likelihood of elevated levels was observed in both cohorts exhibiting clinically significant withdrawal symptoms as well as those with non-clinical levels of withdrawal symptoms. Thus, patients with reported non-clinical levels of withdrawal symptoms may have had a drop in withdrawal scores over time prior to admission when they showed a corresponding drop in adiponectin levels. This observation is consistent with previous reports on heavy drinkers (26) who show a lowering of the withdrawal score over time. This study reported that adiponectin was significantly higher (19.4 μg/ml) in alcohol-dependent patients and that it subsequently started lowering along with the corresponding alcohol withdrawal in the CS groups over the course of observation (26). We find this in our patients, who showed a lowering of adiponectin while maintaining abstinence during inpatient detox. In our study, TNF-α (which was not significantly different from the levels found in our other cohort of AUD patients without withdrawal symptoms (61), Table 2) and HDD90 were associated with AUD patients exhibiting withdrawal symptoms. This supports the unique characterization of proinflammatory activity with a heavy drinking marker indicating a relationship with alcohol-associated neuroinflammation (50, 62), leading to or contributing to withdrawal symptoms (11). Notably, our patients had both chronic (over the years) and recent heavy drinking, which was unique.

### Withdrawal-associated depression in the context of the gut–brain axis

We identified a proinflammatory response originating from gut dysfunction primarily due to LPS (63) and soluble cells of differentiation type 14 (+sCD14) (64) activity in the presentation of symptoms of depression. +sCD14 either appears after the shedding of mCD14 or is directly secreted from intracellular vesicles (65). Immunodepletion of +sCD14 may not be able to prevent inflammatory responses within the blood (66). Thus, the endotoxemia-related macrophageal production of IL-6 (to recruit and engage other cells) and IL-8 (a response to endotoxemia-related defense and increased oxidative stress) can be initiated. Furthermore, IL-8 subsequently facilitates leukocyte transmigration across the blood–brain barrier under both normal (67) and neuropathological conditions (68). Thus, this could facilitate other proinflammatory cytokines reaching CSF and brain tissue. TNF-α response is an early cell-signaling proinflammatory factor that has been reported at lower levels (58) or without significantly different levels (59) in neurological conditions such as depression. These cytokines are capable of crossing the blood–brain barrier (69). IL-6 (60) and IL-8 (70) have been reported to be elevated in AUD patients who reported depression. These two cytokines were observed to play a significant role in the proinflammatory status of the study cohort, especially those who had clinically significant levels of depression (reported by MADRS).

### Gut–brain activity in alcohol craving

Cravings associated with alcohol withdrawal are a well-documented risk factor for subsequent relapse in AUD (21). Many of the neurotransmitters and hormones may regulate and influence the way individuals drink alcohol as an outcome of their response to cravings or other presentations of AUD (71). Leptin is an appetite-regulating hormone produced by adipose, brain, and gut cells and is known for its association with cravings (45). Leptin can be synthesized by the stomach and brain (72, 73), and peripheral leptin can also reach the central nervous system via cellular transportation through the blood-brain barrier (74). Our findings showed a negative relationship between the markers of alcohol intake and leptin, which has also been reported in one previous study (75). Studies have shown that alcohol administration lowered leptin levels in alcohol-preferred rat and mouse model experiments (76, 77). This finding was consistent with our patient population, which reported heavy and chronic drinking. This lowering has been observed in human studies (78) previously. One study supported the diurnal and nocturnal effects of alcohol on leptin concentrations (79), while another suggested that its availability is higher in cerebrospinal fluid (76). We noticed that the women had higher levels of leptin than the men in patients with AUD, regardless of the presence/absence of withdrawal symptoms. This is consistent with recent findings on sex differences in alcohol-drinking behavior (46, 80). Nonetheless, lowering leptin seemed more reflective of heavy drinking in men with AUD (regardless of the presence/absence of withdrawal symptoms) in our study patients.

Cravings are an important domain of AUD that may occur independently of withdrawal symptoms (58). It may occur at non-clinical stages even in social drinkers (81), but how it differs in manifestation when AUD is diagnosed may be explained by the modifying factors that come into play subsequently. Thus, investigating the role of the gut–brain axis specific to craving assessments provided unique reward-associated findings. Our findings shared specific commonalities for precursor gut-permeability factors and proinflammatory cytokine responses with other symptoms. However, subsequent changes in specific metabolites likely contributed to the development of the specific symptoms. Gut dysfunction that is marked by an LBP response coupled with leptin (likely originating from alcohol-associated gut dysfunction) could develop cravings with the ongoing dysregulation of proinflammatory cytokine activity. Hillmer et al. showed that acute alcohol consumption (that could be indicative of cravings or some other presentation of AUD) alters the levels of peripheral cytokines such as IL-8 and TNF-α (82). The proinflammatory activity involved in cravings could originate from the dysregulated cytokine response of both TNF-α and IL-1β, uniquely adhered to in AUD patients with high cravings. As discussed earlier, TNF-α could be involved in triggering the gut-dysfunction-associated immunological dysregulation (83), which perpetuates further with the involvement of IL-1β (84) to uniquely identify the immune signaling response of cravings in AUD patients. Craving, as described by the PACS score in our study, also showed a gut–brain response with the involvement of immune activity. An exogenous phenotype character (behavior responses, clinical assessments) of cravings was highly reproducible with the heavy drinking marker, HDD90, with the same gut–brain axis model. However, the most concerning explanation of such a strong response could present a vicious cycle of the heavy drinking pattern that may be self-feeding to exacerbate through the gut–brain axis.

### Immune dysregulation: relaying gut responses to the brain

Our findings also highlight the elevated levels of IL-6 and IL-8 that are observed with alcohol intake (56). Overproduction of IL-6 [which passes through the blood–brain barrier (57)] and IL-8 [which increases the blood-brain barrier permeability (58)] proceed the initial TNF-α signaling response in patients with AUD, which might not be as contributing to AUD patients without withdrawal symptoms. This could be a major neurobiological key or gateway to withdrawal symptoms (59) and other symptoms of AUD (60), such as withdrawal-associated depression (61). However, how these cytokines may manifest in the domain/s of AUD could be a turning point and, thus, become a highly important area of investigation. Some of these correlations of cytokine levels and the arrangement of gut permeability factors coincide with our earlier observations of hyperhomocysteinemia, gut dysfunctions, and markers of excessive drinking (21). As reflected by the alterations in blood markers, these intestinal changes also lead to behavioral changes that could correspondingly be observed with the severity of alcohol abuse (7, 8, 62). In our study, we found that the derangement of gut-permeability factors and the role of proinflammatory cytokines constituted the gut–brain axis of AUD. Thus, we could identify a three-compartment response system that represents the characterization of the gut–brain axis for such AUD symptoms (with their own uniqueness), with immune status as an intermediate state for neuroinflammatory consequences. These changes were also uniquely representative of withdrawal symptoms and withdrawal-associated depression in a domain-specific manner, along with the gut–brain axis that could represent cravings.

### Limitations in this study

There are some limitations to this study. First, as this was a proof-of-principle study, it had a relatively small sample size. Consequentially, many underlying effects are not addressed in this study and should be the scope of future investigations. NCS patients had only a small sub-group of w (*n* = 5); thus, the interpretations for women are limited. However, the focus was on the patients with AUD and withdrawal symptoms, for which the study included a sufficient number of patients (*N* = 22 [CS] and *N* = 26 [NCS]) to run between-group analyses. Many studies have shown significant elevation of specific cytokines, such as TNF-α, with AUD; however, the current findings support a slight lowering, which we have justified with the available literature suggesting lowering TNF-α.

There is also another explanation for it, which is that with neuroinflammation, once the blood-brain barrier (BBB) has increased permeability, a lot of cytokines influx into the brain. Thus, there may have been a relative lowering of certain cytokines in the blood, an investigation beyond this study's scope. A similar limitation could be associated with leptin levels, where a comparative proportion of the leptin may have crossed the BBB in AUD patients with withdrawal symptoms. Our study was focused on assessing three symptoms in AUD patients with clinically significant domain criteria: CIWA scores, depression ratings, and cravings. Further, we had a subset of patients who reported cravings.

Thus, the assessment of the full cohort was not possible. The direct assessment of reward and reinforcement was not within the scope of this study. We did not see a remarkable interdomain association that could be described conclusively in this study, and it is an area of future research. The identification/role of specific pathogenic gut microbiota was also not investigated. The latter is an area for future investigation. How changes in the various markers might be interrelated and contribute to the different phenotypes detected herein would have been helpful to further support our study. The scope of such findings could be enhanced by fecal metabolomics. We do not have such data in this study, and we hope to analyze the fecal samples in forthcoming studies. PACS was performed only on a subset of participants; thus, the data need to be validated on a larger participant pool in similar studies. We would also like to point out that this is a multi-pronged study, and many foundational findings have been discussed in this investigation. CSF data would be a driver, and we are working on the CSF assessments that will be published to support our findings in subsequent papers.

## Concluding remarks

Gut dysfunction due to heavy alcohol drinking and the gut–brain axis seemed to express the derangement of relevant biomarkers and specific clinical presentation/s in AUD patients in unique arrangements. In this study, we have begun to characterize the gut–brain axis of alcohol withdrawal, associated depression, and cravings, developing a gut–brain model of alcohol pathology with three major realms. The three major realms we have identified are as follows: (1) the direct consequences of drinking on withdrawal marker(s), (2) candidate markers related to gut dysfunction, and (3) specific dysregulation of cytokines as biological compartments of interest. Even though this investigation is a proof-of-principle study, it provides substantial evidence for the pathological differences originating in the gut, spreading up as inflammation, altering the way people feel, and clinically presenting with chronic and heavy drinking. This study also suggests that treating patients with AUD could gain further efficacy with more directed medical management relating to the gut–brain axis. Based on the clinical presentation observed in AUD, the underlying causes can be identified in the gut and immune system. The results of this study warrant the need for further in-depth investigations in the area of the gut–brain axis of AUD.

## Proprietorship

This article is a work of the University of Louisville Alcohol Research Center and the National Institutes of Health. This manuscript is in the public domain in the USA.

## Data availability statement

The original contributions presented in the study are included in the article/Supplementary material, further inquiries can be directed to the corresponding author.

## Ethics statement

The studies involving human participants were reviewed and approved by IRB (University of Louisville) and CNS IRB (NIAAA). The patients/participants provided their written informed consent to participate in this study. The studies were conducted in accordance with the local legislation and institutional requirements. The participants provided their written informed consent to participate in this study.

## Author contributions

VV and CM led this project and designed the study. VV, MLS, HH, and MK participated in the clinical sample and data analyses. VV, RP, AR, MS, MK, HH, MLS, WF, VR, XZ, CM, RA, and JV interpreted the results. VV, RP, MS, HH, MK, VR, XZ, RA, WF, AR, JV, and CM wrote the manuscript. CM, MLS, MK, VR, JV, RA, RP, AR, MS, WF, XZ, and VV critically reviewed the manuscript and contributed scientifically. All authors have approved the submission version of this manuscript.

## References

[1] Kranzler HR Soyka M. Diagnosis and pharmacotherapy of alcohol use disorder: a review. Jama. (2018) 320:815–24. 10.1001/jama.2018.11406 30167705 PMC7391072

[2] Alba-Ferrara L Müller-Oehring E Sullivan E Pfefferbaum A Schulte T. Brain responses to emotional salience and reward in alcohol use disorder. Brain Imaging Behav. (2016) 10:136–46. 10.1007/s11682-015-9374-8 25875013 PMC4607555

[3] McBride WJ Lovinger DM Machu T Thielen RJ Rodd ZA Murphy JM. Serotonin-3 receptors in the actions of alcohol, alcohol reinforcement, and alcoholism. Alcoholism. (2004) 28:257–67. 10.1097/01.ALC.0000113419.99915.DA 15112933

[4] Simon J Etienne A-M Bouchard S Quertemont E. Alcohol craving in heavy and occasional alcohol drinkers after cue exposure in a virtual environment: The role of the sense of presence. Front Human Neurosci. (2020) 14:124. 10.3389/fnhum.2020.00124 32296322 PMC7136534

[5] Anton RF Latham P Voronin K Book S Hoffman M Prisciandaro J. Efficacy of gabapentin for the treatment of alcohol use disorder in patients with alcohol withdrawal symptoms: a randomized clinical trial. JAMA Intern Med. (2020) 180:728–36. 10.1001/jamainternmed.2020.0249 32150232 PMC7063541

[6] Gilpin NW Koob GF. Neurobiology of alcohol dependence: focus on motivational mechanisms. Alcohol Res Health. (2008) 31:185. 19881886 PMC2770186

[7] Leclercq S Matamoros S Cani PD Neyrinck AM Jamar F Stärkel P. Intestinal permeability, gut-bacterial dysbiosis, and behavioral markers of alcohol-dependence severity. Proc Nat Acad Sci. (2014) 111:E4485–E93. 10.1073/pnas.1415174111 25288760 PMC4210345

[8] Leclercq S De Saeger C Delzenne N de Timary P Stärkel P. Role of inflammatory pathways, blood mononuclear cells, and gut-derived bacterial products in alcohol dependence. Biol Psychiatry. (2014) 76:725–33. 10.1016/j.biopsych.2014.02.003 24629538

[9] O'connor J Lawson M Andre C Moreau M Lestage J Castanon N. Lipopolysaccharide-induced depressive-like behavior is mediated by indoleamine 2, 3-dioxygenase activation in mice. Molec Psychiat. (2009) 14:511–22. 10.1038/sj.mp.4002148 18195714 PMC2683474

[10] Kirpich IA McClain CJ Vatsalya V Schwandt M Phillips M Falkner KC. Liver injury and endotoxemia in male and female alcohol-dependent individuals admitted to an alcohol treatment program. Alcoholism. (2017) 41:747–57. 10.1111/acer.13346 28166367 PMC5378598

[11] Gorky J Schwaber J. The role of the gut–brain axis in alcohol use disorders. Progr Neuro-Psychopharmacol Biol Psychiat. (2016) 65:234–41. 10.1016/j.pnpbp.2015.06.013 26188287 PMC4679635

[12] Leclercq S de Timary P Delzenne NM Stärkel P. The link between inflammation, bugs, the intestine and the brain in alcohol dependence. Transl Psychiat. (2017) 7:e1048. 10.1038/tp.2017.15 28244981 PMC5545644

[13] Kelley KW Dantzer R. Alcoholism and inflammation: neuroimmunology of behavioral and mood disorders. Brain Behav Immun. (2011) 25:S13–20. 10.1016/j.bbi.2010.12.013 21193024 PMC4068736

[14] Vatsalya V Gowin JL Schwandt ML Momenan R Coe MA Cooke ME. Effects of varenicline on neural correlates of alcohol salience in heavy drinkers. Int J Neuropsychopharmacol. (2015) 18:pyv068. 10.1093/ijnp/pyv068 26209857 PMC4675979

[15] Bishehsari F Magno E Swanson G Desai V Voigt RM Forsyth CB. Alcohol and gut-derived inflammation. Alcohol Res. (2017) 38:163. 28988571 10.35946/arcr.v38.2.02 PMC5513683

[16] Mayfield J Ferguson L Harris RA. Neuroimmune signaling: a key component of alcohol abuse. Curr Opin Neurobiol. (2013) 23:513–20. 10.1016/j.conb.2013.01.024 23434064 PMC3694992

[17] Vatsalya V Kong M Cave MC Liu N Schwandt ML George DT. Association of serum zinc with markers of liver injury in very heavy drinking alcohol-dependent patients. J Nutr Biochem. (2018) 59:49–55. 10.1016/j.jnutbio.2018.05.003 29960116 PMC6129416

[18] Vatsalya V Hassan HZ Kong M Stangl BL Schwandt ML Schmidt-Teron VY. Exacerbation of Hangover Symptomology Significantly Corresponds with Heavy and Chronic Alcohol Drinking: A Pilot Study. J Clin Med. (2019) 8:1943. 10.3390/jcm8111943 31718086 PMC6912317

[19] Vatsalya V Song M Schwandt ML Cave MC Barve SS George DT. Effects of sex, drinking history, and omega-3 and omega-6 fatty acids dysregulation on the onset of liver injury in very heavy drinking alcohol-dependent patients. Alcoholism. (2016) 40:2085–93. 10.1111/acer.13197 27589090 PMC5367046

[20] Becker HC. Alcohol dependence, withdrawal, and relapse. Alcohol Res Health. (2008) 31:348. 23584009 PMC3860472

[21] Bottlender M Soyka M. Impact of craving on alcohol relapse during, and 12 months following, outpatient treatment. Alcohol Alcoh. (2004) 39:357–61. 10.1093/alcalc/agh073 15208171

[22] Breese GR Sinha R Heilig M. Chronic alcohol neuroadaptation and stress contribute to susceptibility for alcohol craving and relapse. Pharmacol Ther. (2011) 129:149–71. 10.1016/j.pharmthera.2010.09.007 20951730 PMC3026093

[23] Marlatt GA Witkiewitz K. Relapse prevention for alcohol and drug problems. 2005. 10.1037/0003-066X.59.4.224 15149263

[24] Streeton C Whelan G. Naltrexone, a relapse prevention maintenance treatment of alcohol dependence: a meta-analysis of randomized controlled trials. Alcohol Alcohol. (2001) 36:544–52. 10.1093/alcalc/36.6.544 11704620

[25] Loeber S Duka T Welzel Márquez H Nakovics H Heinz A Mann K. Effects of repeated withdrawal from alcohol on recovery of cognitive impairment under abstinence and rate of relapse. Alcohol Alcohol. (2010) 45:541–7. 10.1093/alcalc/agq065 20880959

[26] Buechler C Schäffler A Johann M Neumeier M Köhl P Weiss T. Elevated adiponectin serum levels in patients with chronic alcohol abuse rapidly decline during alcohol withdrawal. J Gastroenterol Hepatol. (2009) 24:558–63. 10.1111/j.1440-1746.2008.05693.x 19067777

[27] Hillemacher T Weinland C Heberlein A Gröschl M Schanze A Frieling H. Increased levels of adiponectin and resistin in alcohol dependence—possible link to craving. Drug Alcohol Depend. (2009) 99:333–7. 10.1016/j.drugalcdep.2008.07.019 18818026

[28] Furtado M Katzman MA. Examining the role of neuroinflammation in major depression. Psychiatry Res. (2015) 229:27–36. 10.1016/j.psychres.2015.06.009 26187338

[29] Troubat R Barone P Leman S Desmidt T Cressant A Atanasova B. Neuroinflammation and depression: a review. Eur J Neurosci. (2021) 53:151–71. 10.1111/ejn.14720 32150310

[30] Hurley LL Tizabi Y. Neuroinflammation, neurodegeneration, and depression. Neurotox Res. (2013) 23:131–44. 10.1007/s12640-012-9348-1 22895696 PMC3751583

[31] Heinz A Beck A Grüsser SM Grace AA Wrase J. Identifying the neural circuitry of alcohol craving and relapse vulnerability. Addict Biol. (2009) 14:108–18. 10.1111/j.1369-1600.2008.00136.x 18855799 PMC2879014

[32] Hartwell EE Ray LA. Craving as a DSM-5 symptom of alcohol use disorder in non-treatment seekers. Alcohol Alcohol. (2018) 53:235–40. 10.1093/alcalc/agx088 29145640

[33] Morris LS Voon V Leggio L. Stress, motivation, and the gut–brain axis: a focus on the ghrelin system and alcohol use disorder. Alcoholism. (2018) 42:1378–89. 10.1111/acer.13781 29797564 PMC6252147

[34] Vatsalya V Gala KS Mishra M Schwandt ML Umhau J Cave MC. Lower serum magnesium concentrations are associated with specific heavy drinking markers, proinflammatory response and early-stage alcohol-associated liver injury. Alcohol Alcohol. (2020) 55:164–70. 10.1093/alcalc/agaa001 32047901 PMC7082490

[35] Vatsalya V Gala KS Hassan AZ Frimodig J Kong M Sinha N. Characterization of early-stage alcoholic liver disease with hyperhomocysteinemia and gut dysfunction and associated immune response in alcohol use disorder patients. Biomedicines. (2021) 9:7. 10.3390/biomedicines9010007 33374263 PMC7823569

[36] Sullivan JT Sykora K Schneiderman J Naranjo CA Sellers EM. Assessment of alcohol withdrawal: the revised clinical institute withdrawal assessment for alcohol scale (CIWA-Ar). Br J Addict. (1989) 84:1353–7. 10.1111/j.1360-0443.1989.tb00737.x 2597811

[37] Müller MJ Szegedi A Wetzel H Benkert O. Moderate and severe depression. Gradations for the montgomery-asberg depression rating scale. J Affect Disord. (2000) 60:137–40. 10.1016/S0165-0327(99)00162-7 10967373

[38] Flannery B Volpicelli J Pettinati H. Psychometric properties of the Penn alcohol craving scale. Alcoholism. (1999) 23:1289–95. 10.1111/j.1530-0277.1999.tb04349.x 10470970

[39] Sobell LC Sobell MB. Timeline follow-back. Measuring alcohol consumption. Totowa, NJ: Humana Press (1992). 41–72 p. 10.1007/978-1-4612-0357-5_3

[40] Skinner HA Sheu W-J. Reliability of alcohol use indices. The lifetime drinking history and the MAST. J Stud Alcohol. (1982) 43:1157–70. 10.15288/jsa.1982.43.1157 7182675

[41] Rosen CS Henson BR Finney JW Moos RH. Consistency of self-administered and interview-based Addiction Severity Index composite scores. Addiction. (2000) 95:419–25. 10.1046/j.1360-0443.2000.95341912.x 10795362

[42] Montgomery SA Åsberg M. A new depression scale designed to be sensitive to change. Br J Psychiat. (1979) 134:382–9. 10.1192/bjp.134.4.382 444788

[43] Yoon G Kim SW Thuras P Grant JE Westermeyer J. Alcohol craving in outpatients with alcohol dependence: rate and clinical correlates. J Stud Alcohol. (2006) 67:770–7. 10.15288/jsa.2006.67.770 16847547

[44] Fukushima K Ueno Y Kawagishi N Kondo Y Inoue J Kakazu E. The nutritional index ‘CONUT' is useful for predicting long-term prognosis of patients with end-stage liver diseases. Tohoku J Exp Med. (2011) 224:215–9. 10.1620/tjem.224.215 21701127

[45] Kiefer F Jahn H Jaschinski M Holzbach R Wolf K Naber D. Leptin: a modulator of alcohol craving? Biol Psychiatry. (2001) 49:782–7. 10.1016/S0006-3223(01)01081-2 11331086

[46] Montague CT Prins JB Sanders L Digby JE O'Rahilly S. Depot- and sex-specific differences in human leptin mRNA expression: implications for the control of regional fat distribution. Diabetes. (1997) 46:342–7. 10.2337/diabetes.46.3.342 9032087

[47] Bode C Bode JC. Effect of alcohol consumption on the gut. Best practice & research. Clin Gastroenterol. (2003) 17:575–92. 10.1016/S1521-6918(03)00034-9 12828956

[48] Bjørkhaug ST Neupane SP Bramness JG Aanes H Skar V Medhus AW. Plasma cytokine levels in patients with chronic alcohol overconsumption: relations to gut microbiota markers and clinical correlates. Alcohol. (2020) 85:35–40. 10.1016/j.alcohol.2019.10.002 31610228

[49] Petrof EO Khoruts A. From stool transplants to next-generation microbiota therapeutics. Gastroenterology. (2014) 146:1573–82. 10.1053/j.gastro.2014.01.004 24412527 PMC4221437

[50] Alfonso-Loeches S Pascual-Lucas M Blanco AM Sanchez-Vera I Guerri C. Pivotal role of TLR4 receptors in alcohol-induced neuroinflammation and brain damage. J Neurosci. (2010) 30:8285–95. 10.1523/JNEUROSCI.0976-10.2010 20554880 PMC6634595

[51] Bajaj JS. Alcohol, liver disease and the gut microbiota. Nat Rev Gastroenterol Hepatol. (2019) 16:235–46. 10.1038/s41575-018-0099-1 30643227

[52] Barki N Bolognini D Jenkins L Hudson B Tobin A Milligan G. P273 Chemogenetic Analysis of How Receptors for Short Chain Fatty Acids Regulate the Gut–Brain Axis. London: BMJ Publishing Group. (2021). 10.1136/gutjnl-2020-bsgcampus.347

[53] Lancaster FE Brown TD Coker KL Elliott JA Wren SB. Sex differences in alcohol preference and drinking patterns emerge during the early postpubertal period in sprague-dawley rats. Alcoholism. (1996) 20:1043–9. 10.1111/j.1530-0277.1996.tb01945.x 8892526

[54] Robbins C. Sex differences in psychosocial consequences of alcohol and drug abuse. J Health Soc Behav. (1989) 30:117–30. 10.2307/2136917 2723378

[55] Agabio R Pisanu C Luigi Gessa G Franconi F. Sex differences in alcohol use disorder. Curr Med Chem. (2017) 24:2661–70. 10.2174/0929867323666161202092908 27915987

[56] Flores-Bonilla A Richardson HN. Sex differences in the neurobiology of alcohol use disorder. Alcohol Res. (2020) 40:4. 10.35946/arcr.v40.2.04 33042719 PMC7532022

[57] Vatsalya V Bin Liaquat H Ghosh K Prakash Mokshagundam SJ McClain C. A review on the sex differences in organ and system pathology with alcohol drinking. Curr Drug Abuse Rev. (2016) 9:87–92. 10.2174/1874473710666170125151410 28124600 PMC5894513

[58] Gowin JL Sloan ME Stangl BL Vatsalya V Ramchandani VA. Vulnerability for alcohol use disorder and rate of alcohol consumption. Am J Psychiatry. (2017) 174:1094–101. 10.1176/appi.ajp.2017.16101180 28774194 PMC5667663

[59] Sierksma A Patel H Ouchi N Kihara S Funahashi T Heine RJ. Effect of moderate alcohol consumption on adiponectin, tumor necrosis factor-α, and insulin sensitivity. Diabetes Care. (2004) 27:184–9. 10.2337/diacare.27.1.184 14693987

[60] Yamauchi T Nio Y Maki T Kobayashi M Takazawa T Iwabu M. Targeted disruption of AdipoR1 and AdipoR2 causes abrogation of adiponectin binding and metabolic actions. Nat Med. (2007) 13:332–9. 10.1038/nm1557 17268472

[61] Zago A Moreira P Jansen K Lhullier AC da Silva RA de Oliveira JF. Alcohol use disorder and inflammatory cytokines in a population sample of young adults. J Alcohol Drug Depend. (2016) 4:2. 10.4172/2329-6488.1000236

[62] Alvarez Cooper I Beecher K Chehrehasa F Belmer A Bartlett SE. Tumour necrosis factor in neuroplasticity, neurogenesis and alcohol use disorder. Brain Plasticity. (2020) 6:47–66. 10.3233/BPL-190095 33680846 PMC7903009

[63] van Eeden WA van Hemert AM Carlier IV Penninx BW Lamers F Fried EI. Basal and LPS-stimulated inflammatory markers and the course of individual symptoms of depression. Transl Psychiat. (2020) 10:235. 10.1038/s41398-020-00920-4 32669537 PMC7363825

[64] Kitchens RL. Role of CD14 in cellular recognition of bacterial lipopolysaccharides. Chem Immunol. (2000) 74:61–82. 10.1159/000058750 10608082

[65] Kirkland T Viriyakosol S. Structure-function analysis of soluble and membrane-bound CD14. Prog Clin Biol Res. (1998) 397:79–87. 9575549

[66] Kitchens RL Thompson PA Viriyakosol S O'Keefe GE Munford RS. Plasma CD14 decreases monocyte responses to LPS by transferring cell-bound LPS to plasma lipoproteins. J Clin Invest. (2001) 108:485–93. 10.1172/JCI200113139 11489942 PMC209364

[67] Hickey WF. Leukocyte traffic in the central nervous system: the participants and their roles. In: Seminars in immunology, Elsevier (1999). 10.1006/smim.1999.0168 10329499

[68] Cross AK Woodroofe MN. Chemokine modulation of matrix metalloproteinase and TIMP production in adult rat brain microglia and a human microglial cell line in vitro. Glia. (1999) 28:183–9. 10.1002/(SICI)1098-1136(199912)28:3<183::AID-GLIA2>3.0.CO;2-3 10559777

[69] Banks WA Kastin AJ Gutierrez EG. Penetration of interleukin-6 across the murine blood-brain barrier. Neurosci Lett. (1994) 179:53–6. 10.1016/0304-3940(94)90933-4 7845624

[70] Kuzior H Fiebich BL Yousif NM Saliba SW Ziegler C Nickel K. Increased IL-8 concentrations in the cerebrospinal fluid of patients with unipolar depression. Compr Psychiatry. (2020) 102:152196. 10.1016/j.comppsych.2020.152196 32927367

[71] Leggio L. Understanding and treating alcohol craving and dependence: recent pharmacological and neuroendocrinological findings. Alcohol Alcohol. (2009) 44:341–52. 10.1093/alcalc/agp026 19451661

[72] Sobhani I Bado A Vissuzaine C Buyse M Kermorgant S Laigneau J. Leptin secretion and leptin receptor in the human stomach. Gut. (2000) 47:178–83. 10.1136/gut.47.2.178 10896907 PMC1727985

[73] Ur E Wilkinson DA Morash BA Wilkinson M. Leptin immunoreactivity is localized to neurons in rat brain. Neuroendocrinology. (2002) 75:264–72. 10.1159/000054718 11979057

[74] Banks WA Kastin AJ Huang W Jaspan JB Maness LM. Leptin enters the brain by a saturable system independent of insulin. Peptides. (1996) 17:305–11. 10.1016/0196-9781(96)00025-3 8801538

[75] Wayne S Neuhouser ML Ulrich CM Koprowski C Wiggins C Baumgartner KB. Association between alcohol intake and serum sex hormones and peptides differs by tamoxifen use in breast cancer survivors. Cancer Epidemiol Prev Biomark. (2008) 17:3224–32. 10.1158/1055-9965.EPI-08-0171 18957523 PMC2673729

[76] Mikolajczak P Okulicz-Kozaryn I Kamiñska E Wiktorowicz K Lesniewska K Dyr W. Effect of subchronic ethanol treatment on plasma and cerebrospinal fluid leptin levels in rats selectively bred for high and low alcohol preference. Pol J Pharmacol. (2002) 54:127–32. 12139109

[77] Tan X Sun X Li Q Zhao Y Zhong W Sun X. Leptin deficiency contributes to the pathogenesis of alcoholic fatty liver disease in mice. Am J Pathol. (2012) 181:1279–86. 10.1016/j.ajpath.2012.06.013 22841822 PMC3463622

[78] Santolaria F Pérez-Cejas A Alemán M-R González-Reimers E Milena A De La Vega M-J. Low serum leptin levels and malnutrition in chronic alcohol misusers hospitalized by somatic complications. Alcohol Alcohol. (2003) 38:60–6. 10.1093/alcalc/agg015 12554610

[79] Röjdmark S Calissendorff J Brismar K. Alcohol ingestion decreases both diurnal and nocturnal secretion of leptin in healthy individuals. Clin Endocrinol. (2001) 55:639–47. 10.1046/j.1365-2265.2001.01401.x 11894976

[80] Bouna-Pyrrou P Muehle C Kornhuber J Weinland C Lenz B. Body mass index and serum levels of soluble leptin receptor are sex-specifically related to alcohol binge drinking behavior. Psychoneuroendocrinology. (2021) 127:105179. 10.1016/j.psyneuen.2021.105179 33780690

[81] Stangl BL Vatsalya V Zametkin MR Cooke ME Plawecki MH O'Connor S. Exposure-response relationships during free-access intravenous alcohol self-administration in nondependent drinkers: influence of alcohol expectancies and impulsivity. Int J Neuropsychopharmacol. (2017) 20:31–9. 10.1093/ijnp/pyw090 27742833 PMC5412584

[82] Hillmer AT Nadim H Devine L Jatlow P O'Malley SS. Acute alcohol consumption alters the peripheral cytokines IL-8 and TNF-α. Alcohol. (2020) 85:95–9. 10.1016/j.alcohol.2019.11.005 31759072 PMC7739954

[83] Heberlein A Käser M Lichtinghagen R Rhein M Lenz B Kornhuber J. TNF-α and IL-6 serum levels: neurobiological markers of alcohol consumption in alcohol-dependent patients? Alcohol. (2014) 48:671–6. 10.1016/j.alcohol.2014.08.003 25262503

[84] Airapetov M Eresko S Lebedev A Bychkov E Shabanov P. The role of Toll-like receptors in neurobiology of alcoholism. Biosci Trends. (2021) 15:74–82. 10.5582/bst.2021.01041 33716257

