# Supplementary material for: Novel paradigms for the gut–brain axis during alcohol withdrawal, withdrawal-associated depression, and craving in patients with alcohol use disorder
Source: Front Psychiatry. 2023 Sep 29;14:1203362. doi: 10.3389/fpsyt.2023.1203362 (PMC10570744; doi:10.3389/fpsyt.2023.1203362)
Supplement: Supplementary file 1 [file Data_Sheet_1.pdf]

Title: Novel Paradigms for the Gut-Brain Axis of Alcohol Withdrawal, Withdrawal-Associated Depression, and Craving in Patients with Alcohol Use Disorder

Authors: Vatsalya Vatsalya[1,2,3,4,*,#], Joris C. Verster[5,6], Manasa Sagaram[1,4], Amor J. Royer[1,4], Huirong Hu[4,7], Ranganathan Parthasarathy[4], Melanie L. Schwandt[3], Maiying Kong[7,8], Vijay A Ramchandani[3], Wenke Feng[1,8,9,10], Ruchita Agrawal[11], Xiang Zhang[8,9,10,12,13], Craig J. McClain[1,2,7,8,10,13#]

Affiliations:
1Division of Gastroenterology, Hepatology and Nutrition, Department of Medicine, University of Louisville, Louisville, KY, United States,
2Robley Rex VA Medical Center, Louisville, KY, United States,
3National Institute on Alcohol Abuse and Alcoholism, Bethesda, MD, United States,
4Clincial Laboratory for the Intervention Development of AUD and Organ Severity, Louisville, KY, United States,
5Utrecht Institute for Pharmaceutical Sciences (UIPS), Utrecht University, Utrecht, Netherlands,
6Centre for Human Psychopharmacology, Swinburne University, Melbourne, VIC, Australia,
7Department of Bioinformatics and Biostatistics, University of Louisville, Louisville, KY, United States,
8Alcohol Research Center, University of Louisville, Louisville, KY, United States,
9Department of Pharmacology & Toxicology, University of Louisville, Louisville, KY, United States,
10Hepatobiology & Toxicology Center, University of Louisville, Louisville, KY, United States,
11Seven Counties, Louisville, KY, United States,
12Department of Chemistry, University of Louisville, Louisville, KY, United States,
13Center for Regulatory and Environmental Analytical Metabolomics, University of Louisville, Louisville, KY, United States

# These two authors share equal senior authorship

Supplementary Material

Results

Alcohol Addiction Severity Index

Alcohol Composite Score (ACS, another measure of alcohol drinking that describes the Addiction Severity Index) and HDD90 measures were significantly and positively associated (R^2=0.318 at p≤0.001) as measure of heavy drinking consequential in severity due to alcohol drinking across all the patients (S. Fig 1a). ACS was also found to be significantly associated with

the gut permeability markers, Lipopolysaccharide (LPS), and Lipid binding protein (LBP) respectively across all the patients (S. Fig 1b-1c).

Discussion

AUD patients show a corresponding positively related response by the degree of heavy drinking in recent active past and the severity index of alcohol drinking as reported by ACS [1]. Our evaluation supports a pathological connection of the correspondingly increasing order of the severity index of alcohol drinking and LPS (an altered gut dysfunction response characterized by the alcohol associated endotoxemia [2]). Elevated LBP with low ACS could play a enhancing role, albeit lower LBP at higher ACS could well be directed towards decreasing the biological activity of LPS in excess of LPS [3], as observed with a negative relationship in our findings. A valid questionnaire such as ACS based assessment could present a valuable link between the recent heavy drinking pattern and the gut-dysregulation that is encountered in the AUD patients.

Supplementary Figure Legend

Supplementary Figure Legend 1. Severity of alcohol drinking, and its associaiton with alcohol drinking pattern and gut dysregulation in all AUD patients. Fig. S1a. Heavy drinking days in the past 90-day (HDD90) showed high positive relationshsip with the alcohol composite acore (ACS). Fig. S1b. ACS showed significant positive associaiton with the lipopolysaccaride. Fig. S1c. ACS showed significant negative associaiton with the lipopolysaccaride-binding protein (LBP). Statistical significance was set at $p<0.05$.

References

1. Rosen CS, Henson BR, Finney JW, Moos RH. Consistency of self-administered and interview-based Addiction Severity Index composite scores. *Addiction.* 2000;95(3):419-425.
2. Kirpich IA, McClain CJ, Vatsalya V, et al. Liver injury and endotoxemia in male and female alcohol-dependent individuals admitted to an alcohol treatment program. *Alcoholism: Clinical and Experimental Research.* 2017;41(4):747-757.
3. Lamping N, Dettmer R, Schröder N, et al. LPS-binding protein protects mice from septic shock caused by LPS or gram-negative bacteria. *The Journal of clinical investigation.* 1998;101(10):2065-2071.
