# Supplementary figures and images for: Novel paradigms for the gut–brain axis during alcohol withdrawal, withdrawal-associated depression, and craving in patients with alcohol use disorder
Source: Front Psychiatry. 2023 Sep 29;14:1203362. doi: 10.3389/fpsyt.2023.1203362 (PMC10570744; doi:10.3389/fpsyt.2023.1203362)

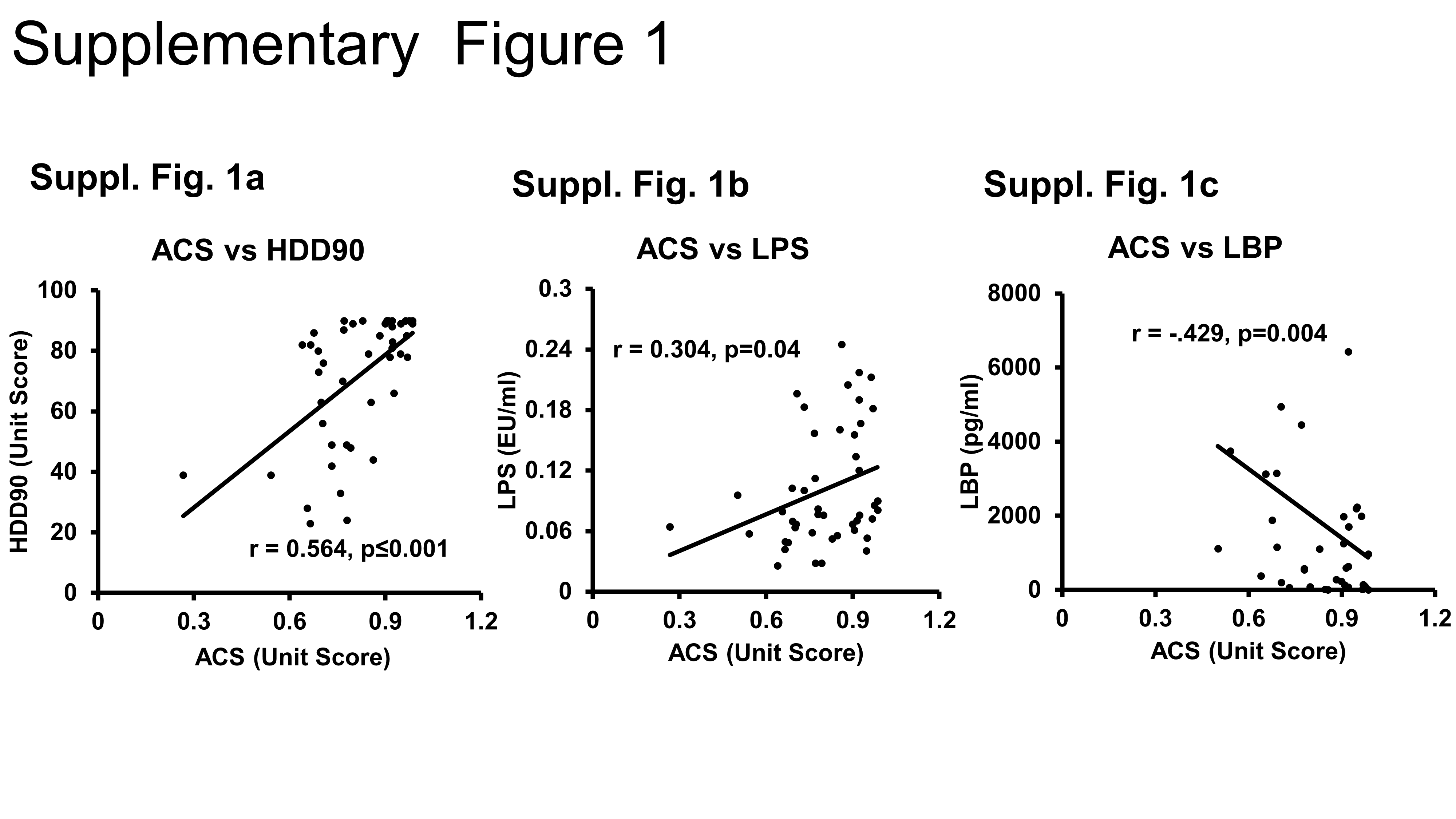

Supplement: Supplementary file 2 [file Image_1.TIF]
